# Supplementary material for: Tyrosyl-tRNA synthetase has a noncanonical function in actin bundling
Source: Nat Commun. 2023 Mar 8;14:999. doi: 10.1038/s41467-023-35908-3 (PMC9995517; doi:10.1038/s41467-023-35908-3)
Supplement: Supplementary file 1 — Supplementary Information [file 41467_2023_35908_MOESM1_ESM.docx]

**Tyrosyl-tRNA synthetase has a non-canonical function in actin bundling**

Biljana Ermanoska^1,2,3^, Bob Asselbergh^4,5^, Laura Morant^1,2^, Maria-Luise Petrovic-Erfurth^1,2^, Seyyedmohsen Hosseinibarkooie^6†^, Ricardo Leitão-Gonçalves^1,2‡^, Leonardo Almeida-Souza^1,2§^, Sven Bervoets^1,2¶^, Litao Sun^7#^, LaTasha Lee^8**^, Derek Atkinson^1,2††^, Akram Khanghahi^1, 2^, Ivaylo Tournev^9,10^, Patrick Callaerts^11^, Patrik Verstreken^12,13^, Xiang-Lei Yang^7^, Brunhilde Wirth^6^, Avital A. Rodal^3^, Vincent Timmerman^2^, Bruce L. Goode^3^, Tanja A. Godenschwege^8^, and Albena Jordanova^1,2,14*^

**Affiliations:**

^1^ Center for Molecular Neurology, VIB, University of Antwerp, 2610 Antwerpen, Belgium.

^2^ Department of Biomedical Sciences, University of Antwerp, 2610 Antwerpen, Belgium.

^3^ Department of Biology, Brandeis University, Waltham, MA 02453, USA.

^4^ Neuromics Support Facility, VIB Center for Molecular Neurology, VIB, 2610 Antwerp, Belgium

^5^ Neuromics Support Facility, Department of Biomedical Sciences, University of Antwerp, 2610 Antwerp, Belgium

^6^ Institute of Human Genetics; Center for Molecular Medicine Cologne; Center for Rare Diseases Cologne; University Hospital of Cologne; University of Cologne, 50931 Cologne, Germany.

^7^ Department of Molecular Medicine, The Scripps Research Institute, La Jolla, CA 92037, USA.

^8^ Department of Biological Sciences, Florida Atlantic University, Jupiter, FL 33458, USA.

^9^ Department of Neurology, Medical University-Sofia, 1431 Sofia, Bulgaria.

^10^ Department of Cognitive Science and Psychology, New Bulgarian University, 1618 Sofia, Bulgaria.

^11^ Department of Human Genetics, KU Leuven, 3000 Leuven, Belgium.

^12^ VIB-KU Leuven Center for Brain & Disease Research, 3000 Leuven, Belgium

^13^ KU Leuven, Department of Neurosciences, Leuven Brain Institute, Mission Lucidity, 3000 Leuven, Belgium

^14^ Department of Medical Chemistry and Biochemistry, Medical University-Sofia, 1431 Sofia, Bulgaria.

**This supplementary information file includes:**

Supplementary Figures 1 to 11

References for the supplementary information

**Other supplementary materials for this manuscript include the following:**

Supplementary Movie 1

Supplementary Movie 2

Supplementary Data 1. X-chromosome-associated *EP* lines used for a retinal degeneration screen in YARS1^E196K^ *Drosophila* model.

Supplementary Data 2. Table of mass-spectrometry data in FLAG-YARS1^WT^ expressing HEK293 cells.


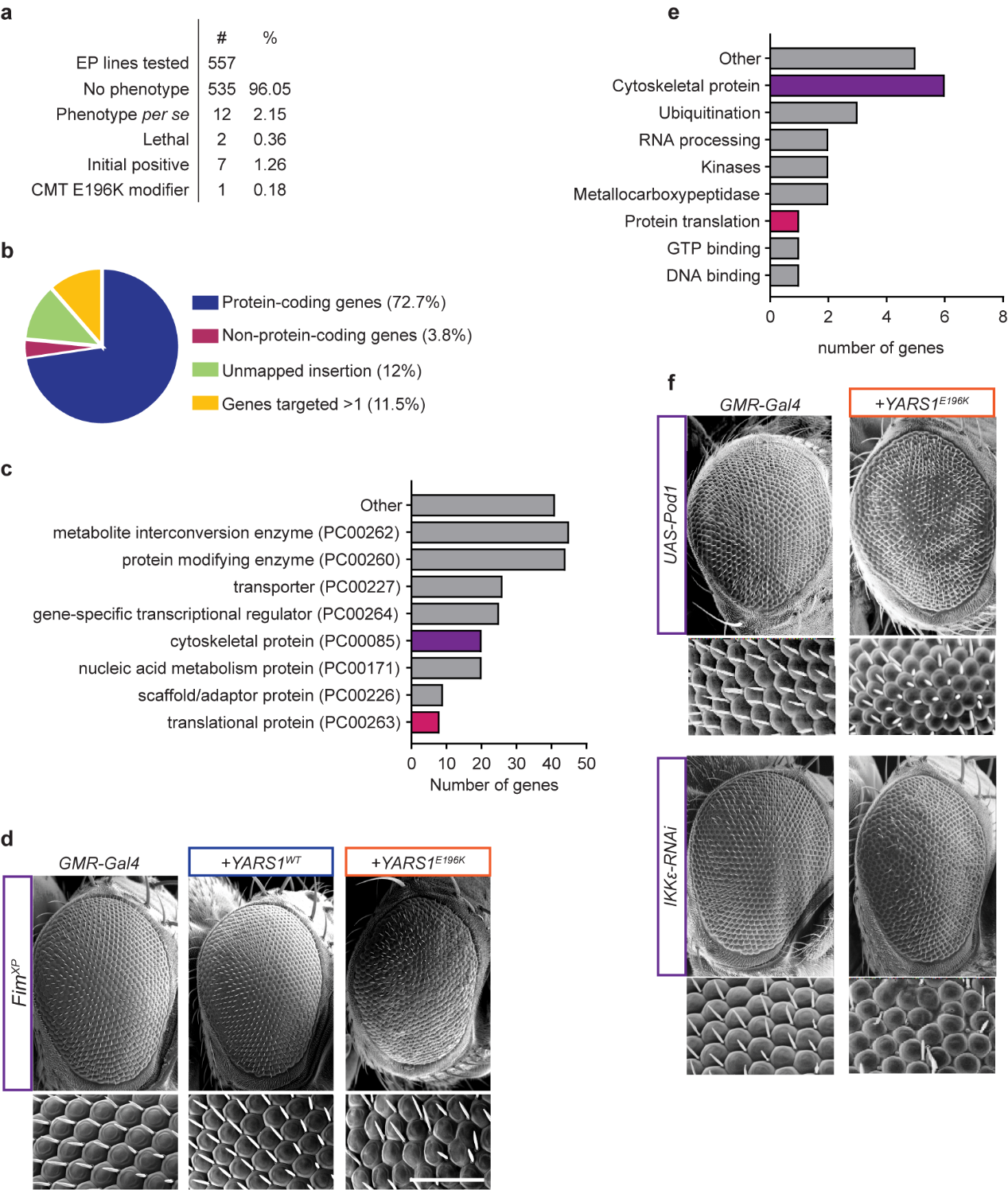


**Supplementary Figure 1. Retinal degeneration screen in YARS1^CMT^ *Drosophila*.**

**(a)** An overview of phenotypes in the retinal degeneration genetic screen shows that 96% of the individual *EP* lines scored had no obvious eye phenotype when missexpressed with the *GMR-Gal4* driver. About 2% presented some extent of an eye defect when expressed alone and were not considered for further assessment, and 2 lines even exhibited lethality upon *GMR-Gal4* missexpression. Seven *EP* lines were initially discovered to induce a rough eye phenotype when co-expressed with *YARS1^E196K^*, but after subsequent validation (described in detail in the Methods) only *Fim^EP^* matched the criteria of being a *YARS1^E196K^* modifier. (**b)** An overview of the genomic location of the 557 *EP* lines shows that a large fraction (72.7%) targeted protein-coding genes. EP insertions in- or proximal to non-protein coding genes (long-noncoding RNAs, anti-sense coding RNAs, pseudogenes, unannotated genes) comprised 3.4%, while 12% were unmapped. Most of the *EPs* targeted unique genomic regions. (**c)** PANTHER-based functional classification demonstrates that the proteins encoded by the genes potentially targeted by the *EP* lines belong to different functional categories. (**d)** Scanning electron micrographs of adult fly eyes in animals co-expressing an independent *Fim^XP^* line with *YARS1^WT^* and *YARS1^E196K^*. (**e)** Twenty-three additional genes, belonging to different functional classes and retrieved as putative interactors from the interaction networks of YARS1 and Fim listed in DroID ^1^, were analyzed for enhancement of *YARS1^E196K^* ommatidial disorganization. (**f)** *GMR-Gal4*-driven over-expression of the actin-related protein dPod1 and down-regulation of the IKKε kinase, known to regulate F-actin assembly in *Drosophila*, induced ommatidial disorganization when co-expressed with *YARS1^E196K^*. The experiments were repeated at least three independent times with similar outcomes. Source data are provided as a Source data file.


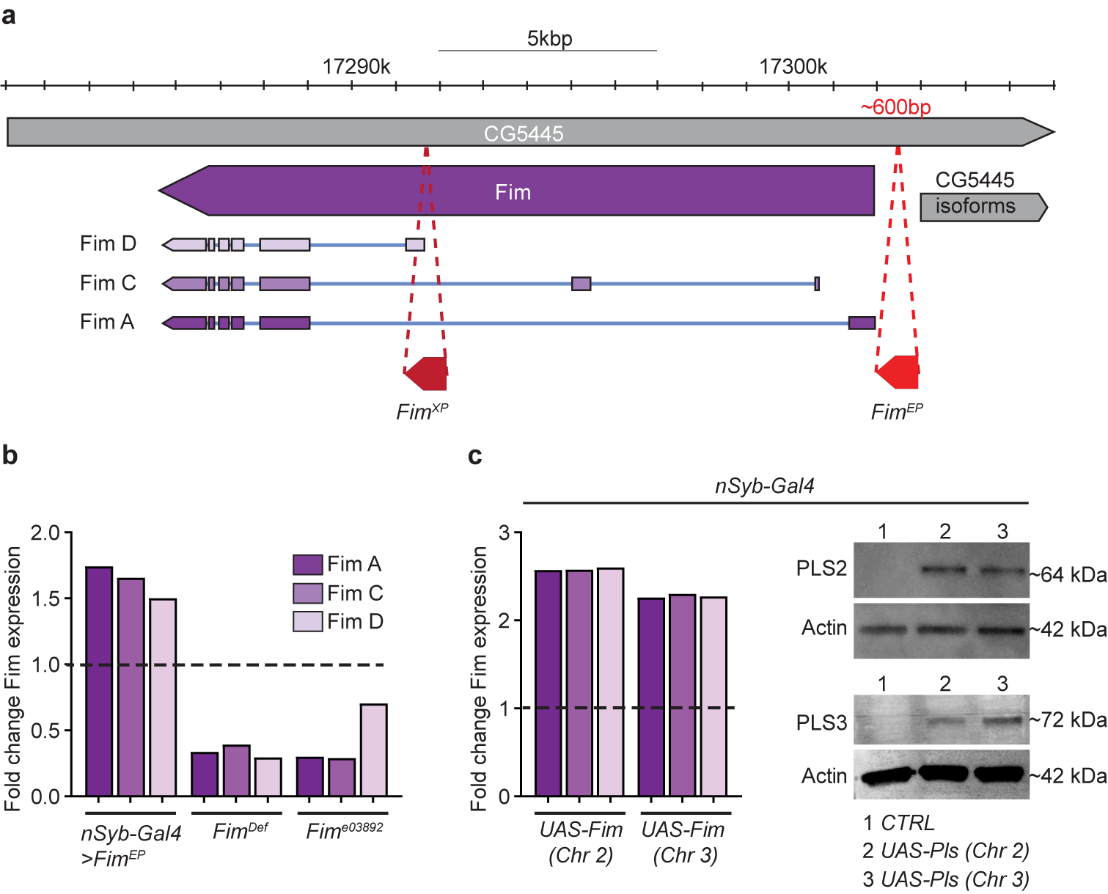


**Supplementary Figure 2. Molecular characterization of Fim alleles.**

**(a)** Schematic representation of the genomic region of Fimbrin (CG8649/Fim) and its three predicted isoforms (*Fim A*, *Fim C* and *Fim D*), on the *D. melanogaster* X-chromosome. The bright red arrow indicates the 5’-end of *Fim^EP^*, recovered by iPCR remapping, confirming single genomic insertion as previously annotated. We confirmed by qPCR that the expression level of the overlapping gene CG5445 is unaffected by the *Fim^EP^* insertion (data not shown). The insertion of the independent *Fim^XP^* line, that mildly enhances the ommatidial disorganization of *YARS1^E196K^*-expressing flies, is depicted with dark red arrow. (**b)** Graph shows the fold change in mRNA levels of the three Fim isoforms upon *nSyb-Gal4*-driven expression of *Fim^EP^*, normalized to the expression level of control (*nSyb-Gal4/+*) flies (dashed line), assessed by qPCR. In the same graph we present the relative decrease of expression of Fim isoforms in the deficiency line *Fim^Def^* and the *Fim^e03892^* hypomorph. The experiment was repeated twice, with independent RNA isolations of flies from independent crosses. **c** Expression level analysis of *UAS-Fim*, *UAS-PLS3* and *UAS-PLS2* upon *nSyb-Gal4*-targeted expression of independent transgenic lines, inserted on the second and third chromosomes. mRNA levels of Fim isoforms were analyzed by qPCR in the *nSyb>Gal4>UAS-Fim* flies, while the expression levels of PLS3 and PLS2 were assessed by Western blot of adult head protein extracts of flies expressing the respective transgenes pan-neuronally (*nSyb-Gal4*). Control in both the qPCR and the Western blot analyses are *nSyb-Gal4/+* flies. The experiments were repeated at least two independent times with similar outcomes. Source data are provided as a Source data file.


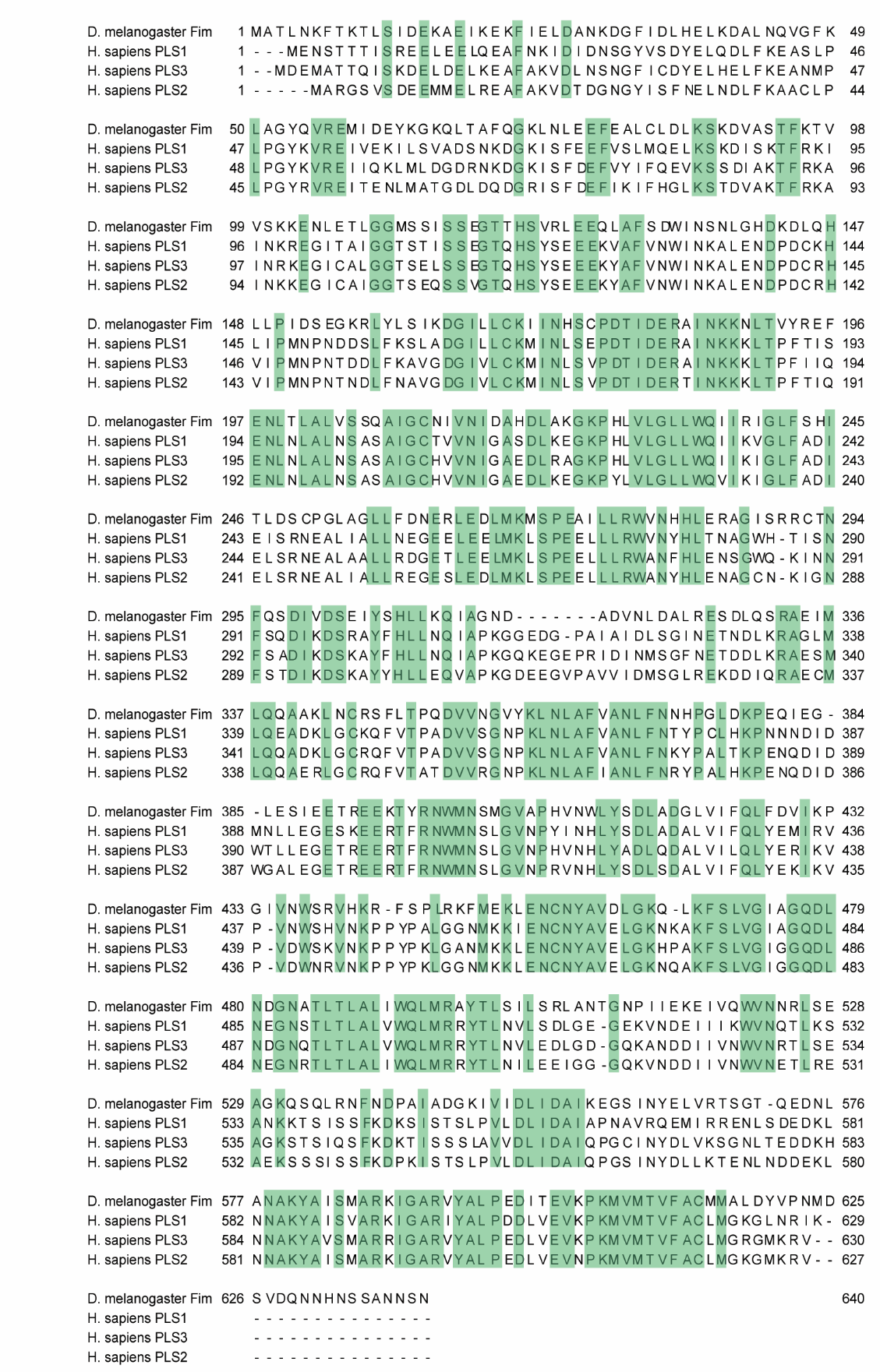


**Supplementary Figure 3. Protein sequence alignment of the *Drosophila* Fimbrin and the three human orthologues PLS3, PLS2 and PLS1.**

The human Plastin isoforms share 85% identity between each other and 57% identity with Fimbrin, as determined by NCBI Blast. Green denotes identical residues. ClustalOmega software ^2^ was used to perform the sequence alignment. The different Plastin isoforms have cell-type-specific expression: PLS3 is expressed in cells from solid tissues, such as neurons, PLS2 is expressed in hematopoietic cell lineages, and PLS1 is restricted to intestine and kidney ^3^. Based on the expression pattern and its relevance as a genetic modifier in spinal muscular atrophy ^4,5^, we chose to generate transgenic flies carrying *PLS3*, and *PLS2*.


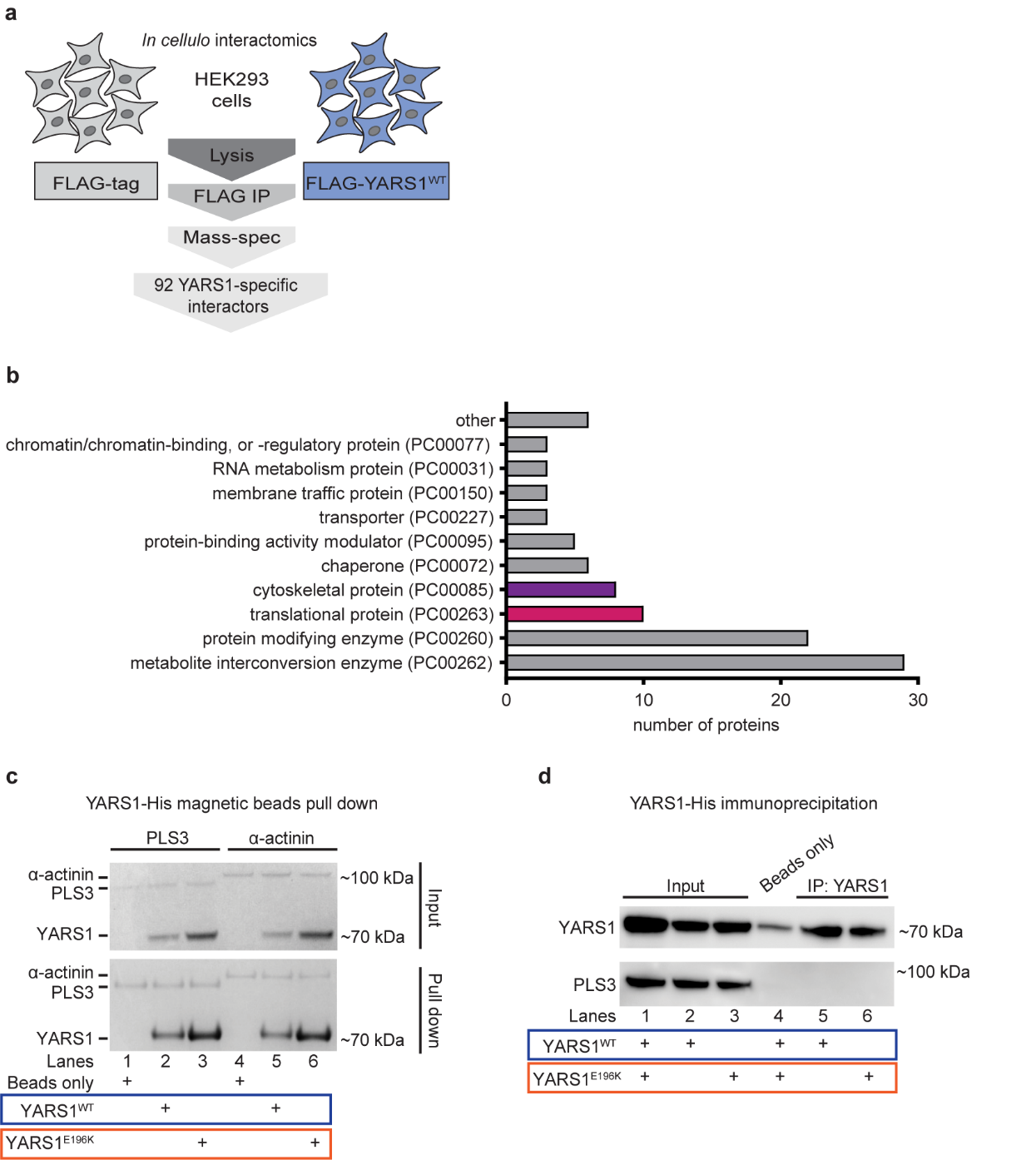


**Supplementary Figure 4. YARS1^WT^ interactors identified via FLAG-based immunoprecipitation in mammalian cells.** (**a)** A scheme of the *in cellulo* approach, where human YARS1^WT^ interactors were co-purified from HEK293 cells overexpressing FLAG-YARS1^WT^ by immunoprecipitation with subsequent mass-spectrometry. **(b)** PANTHER-based molecular function classification of proteins that co-purified with FLAG-tagged YARS1^WT^ (but not control FLAG only), including 8 cytoskeletal proteins and 10 proteins with a function in protein translation. See also Supplementary Data 2. **(c)** Magnetic beads pull down of recombinant His-tagged YARS1, incubated with PLS3 (lanes 2 and 3) or α-actinin (lanes 5 and 6) does not support a direct interaction with either of the two proteins. (**d)** Immunoprecipitation of recombinant His-tagged YARS1 incubated with recombinant PLS3 confirms the lack of direct interaction between YARS1 and PLS3. Source data are provided as a Source data file.


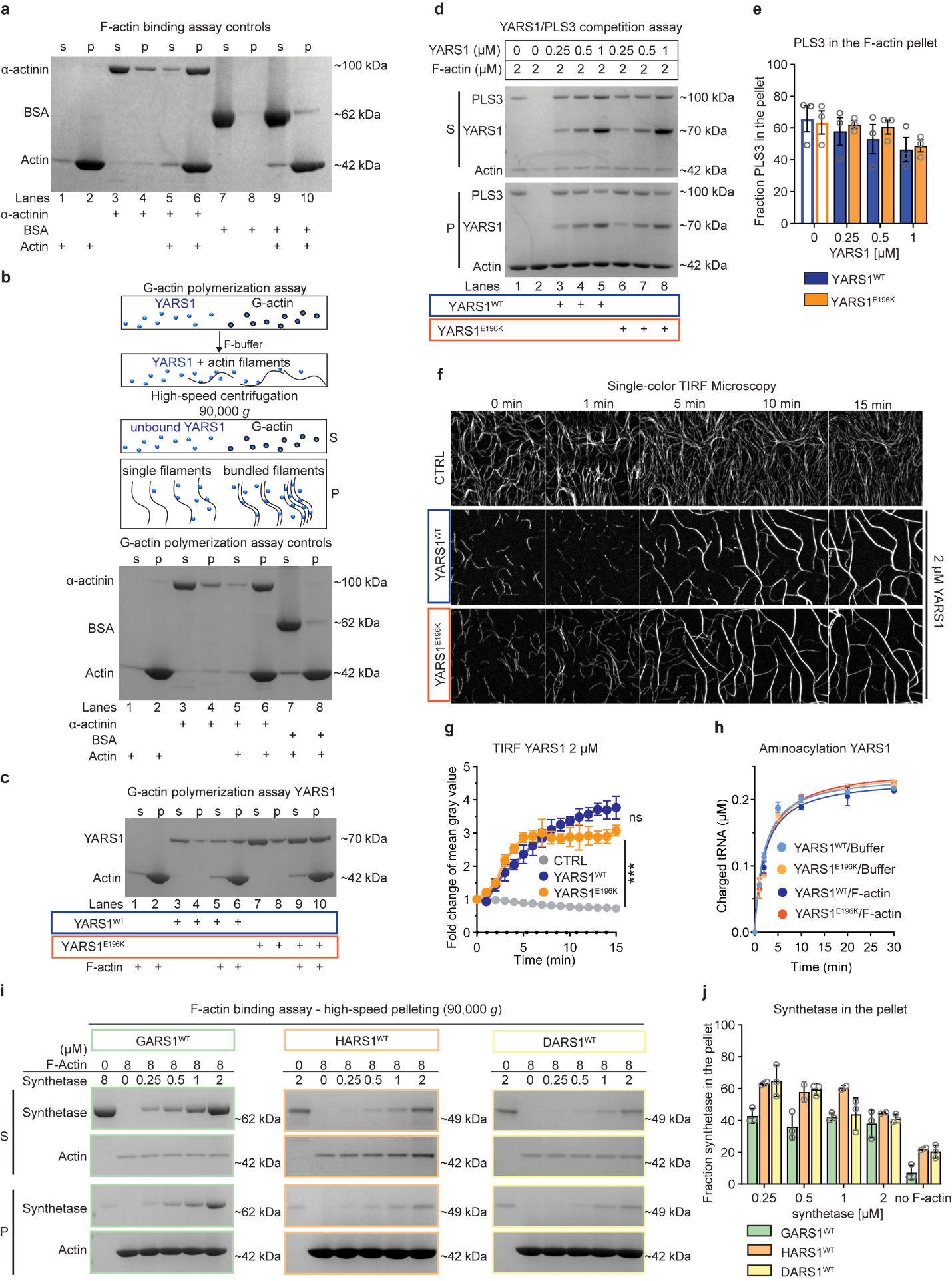


**Supplementary Figure 5. *In vitro* assessment of protein-protein interactions.** (**a)** High-speed pelleting assay to assess the F-actin binding of α-actinin (an established actin binding and bundling protein) as a positive control and BSA (non-actin binding) as a negative control. Residual F-actin is found in the supernatants (s) (lanes 1, 5 and 9), and most of F-actin is found in the pellets (p) (lanes 2, 6 and 10). Soluble α-actinin in the absence of F-actin is mostly detected in the supernatant (lanes 3 and 4) but shifts to the pellet when co-incubated with F-actin (lanes 5 and 6). On contrary, BSA remains in the supernatant regardless of the absence (lanes 7 and 8), or presence of F-actin (lanes 9 and 10). (**b)** Assessment of *de novo* polymerization of monomeric G-actin. Polymerization of G-actin is initiated by adding F-buffer, waiting for the reaction to reach equilibrium for 1 hour, followed by high-speed pelleting. The G-actin to F-actin polymerization without additional proteins (lanes 1 and 2), in the presence of α-actinin (lanes 5 and 6) or BSA (lanes 7 and 8). (**c)** *De novo* F-actin polymerization is unperturbed on its own (lanes 1 and 2), or in presence of YARS1^WT^ (lanes 5 and 6) and YARS1^E196K^ (lanes 9 and 10). The shift of the YARS1 proteins from the supernatant to the pellet in the *de novo* actin polymerization reaction is comparable to the shift when YARS1 is incubated with pre-assembled F-actin (Fig. 2a). The experiments were repeated two independent times with similar outcomes. (**d)** High speed pelleting assay upon co-incubation of 2 μM F-actin with 1 μM PLS3 and YARS1 at three different concentrations demonstrates a mild decrease of the actin-binding and bundling protein PLS3 from the pellet to the supernatant in presence of YARS1, as plotted in (**e)**, where *n* = 3 independent co-sedimentation assays. Supernatant and pellet samples in **a**, **b**, **c**, and **d** derive from the same experiment and gels were processed in parallel. (**f)** Representative time-lapse images from a single color TIRF microscopy of 10% OG-actin labeled filaments alone or in the presence of 2 μM YARS1 demonstrates strong bundling and actin cables formation. Scale bar – 20 μm. (**g)** The fold change of mean gray value of the fluorescent OG-actin signal increased significantly from the moment of flow-in of 2 μM YARS1, as a result of the actin bundles and cables formation, unlike the lack of bundle formation in the control conditions upon buffer only flow-in. *n* = 3 represents randomly selected, non-overlapping fields-of-view, imaged within one TIRF experiment per condition (CTRL, YARS1^WT^, and YARS1^E196K^). Error bars – SD. *** p=0.0002, two-sided unpaired t-test. (**h)** Aminoacylation activity of YARS1^WT^ and YARS1^E196K^, assessed via measurement of the charged tRNA over time, is unaffected in absence or presence of F-actin at 1:4 molar ratio; *n* = 2 technical replicates from one out of two independent experiments. (**i)** High speed pelleting assay upon co-incubation of 8 μM preassembled F-actin with GARS1, HARS1 and DARS1 at three different concentrations demonstrates that these synthetases also bind to F-actin. Supernatant and pellet samples derive from the same experiment and gels were processed in parallel. (**j)** A graph representing the fraction of GARS1, HARS1 and DARS1 bound to the F-actin pellet, where *n* = 3 independent co-sedimentation assays for GARS1 and DARS1, and *n* = 2 for HARS1. Data in **e**, **h** and **j** presented as mean values +/- SEM. Source data are provided as a Source data file.


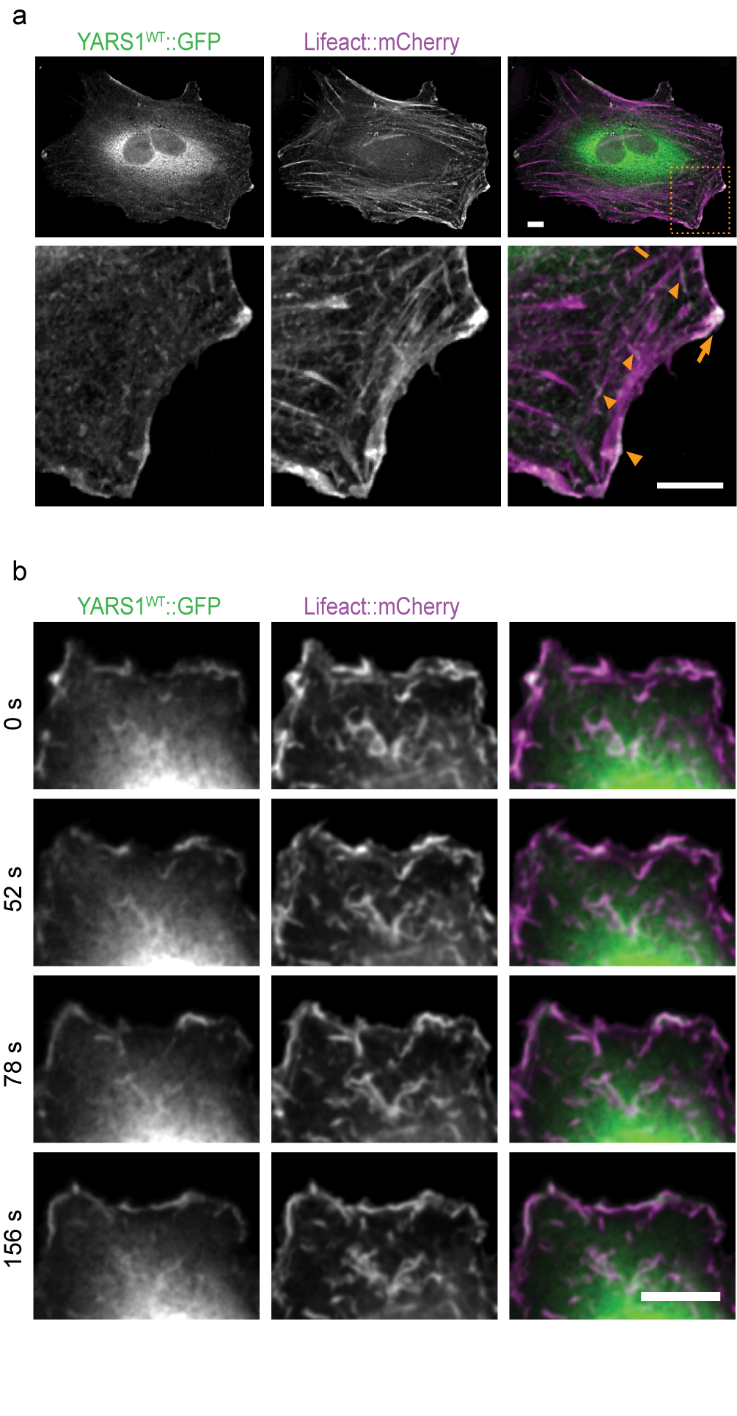


**Supplementary Figure 6. Visualization of YARS1^WT^-eGFP and Lifeact-labeled F-actin in HeLa cells.** (**a)** YARS1^WT^::eGFP is present at the F-actin (Lifeact::mCherry) cell cortex (arrows) and at short transverse F-actin fibers (arrowheads). (**b)** Time lapse imaging of living cells shows overlapping YARS1^WT^::eGFP and Lifeact::mCherry at the dynamic border regions of the cell. The experiments were repeated at least three independent times with similar outcomes. See also the corresponding full time lapse movie as Supplementary movie 2. Scale bar – 10 µm.


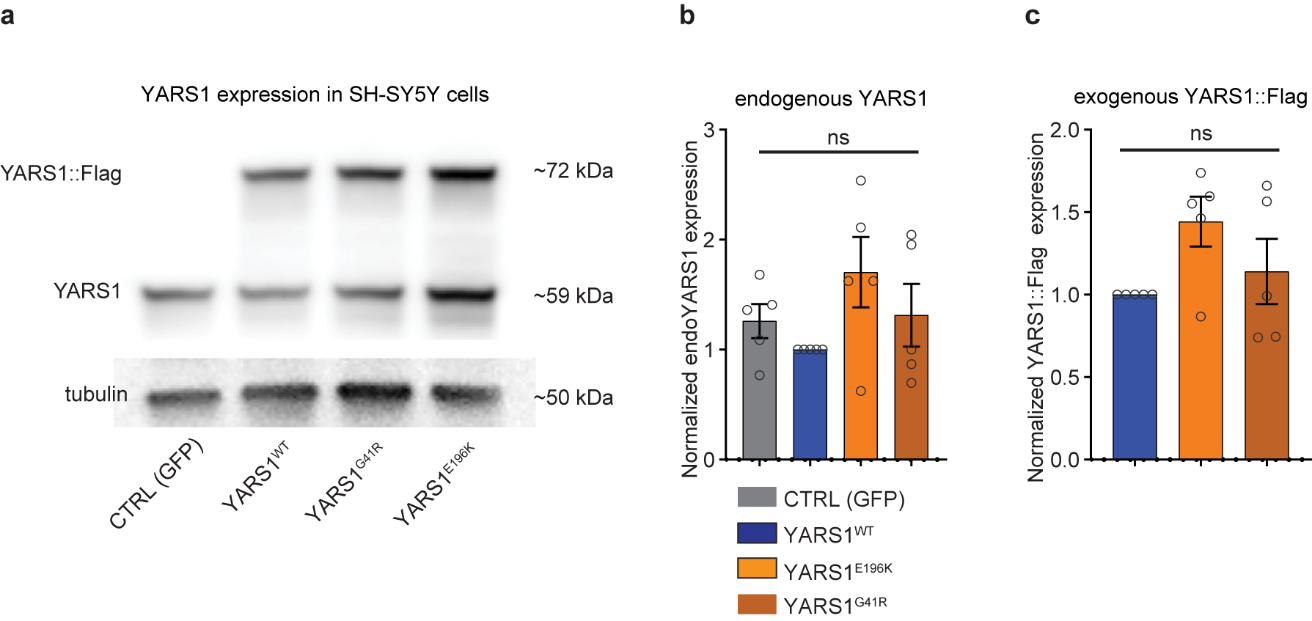


**Supplementary Figure 7. Assessment of YARS1 expression levels in** SH-SY5Y neuroblastoma cells stably expressing YARS1 transgenes**. (a)** Representative Western blot of SH-SY5Y cell extracts from control- and lines expressing FLAG-tagged YARS1^WT^ and two CMT mutants. **(b)** Quantification of the relative expression levels of endogenous YARS1. **(c)** Quantification of the relative expression levels of exogenous FLAG-tagged YARS1. *n* = 5 independent Western blot experiments; ns – nonsignificant, with a one-way Anova statistical analysis to compare YARS1 protein expression levels. Data in **b** and **c** are presented as as mean values +/- SEM. Source data are provided as a Source data file.

**
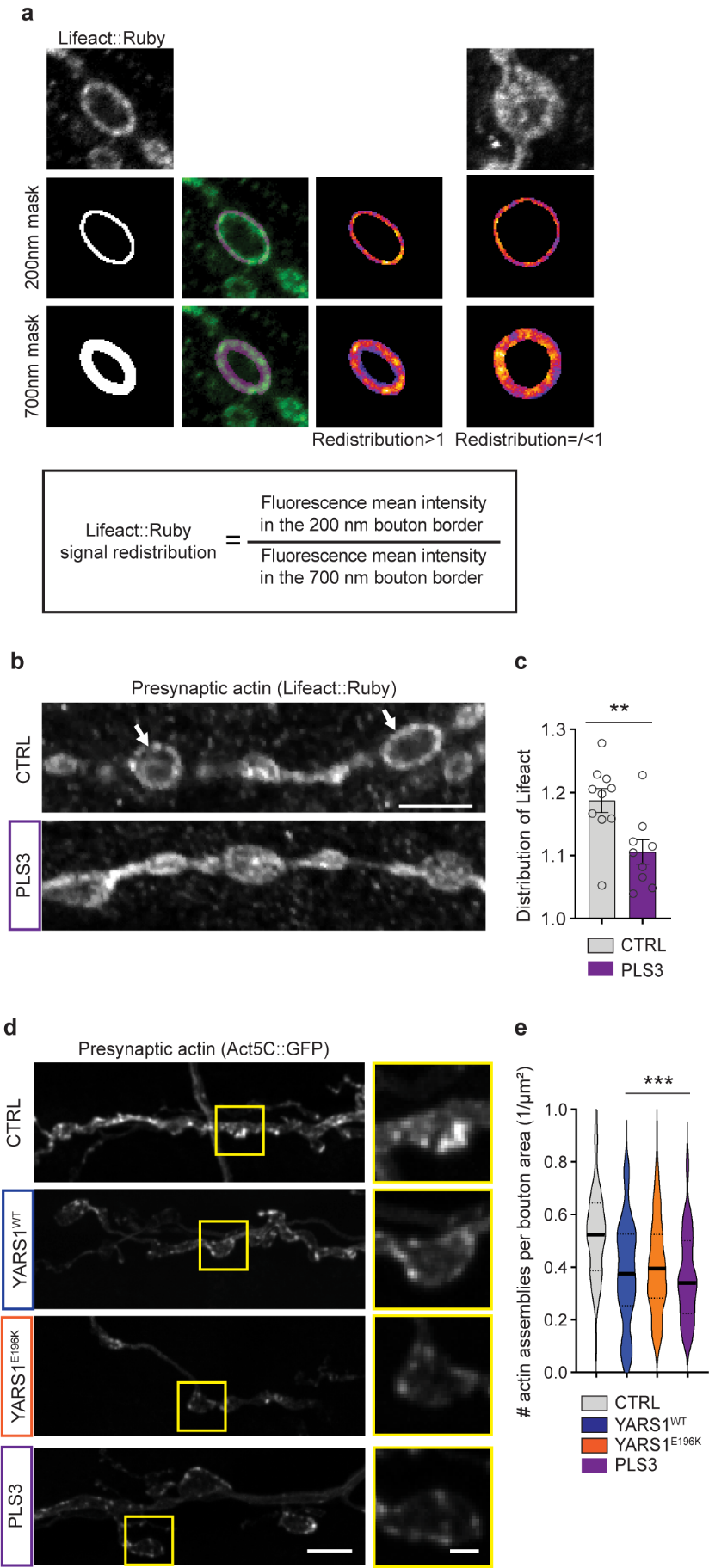
**

**Supplementary Figure 8. Quantitative analysis of the actin cytoskeleton at the larval NMJ.**

**(a)** Illustration of the quantification procedure to measure the presynaptic distribution of the Lifeact fluorescence signal towards the bouton border. To measure how much signal is confined in the outer thin rim of the bouton versus a broader, more distributed rim, the intensity ratio between these two zones was measured. The bouton border is manually delineated with border masks of 200 nm or 700 nm thickness, and the fluorescence mean intensity of Lifeact::Ruby is measured within the two masks, using an ImageJ script. Two example boutons are shown: on left one with a redistribution >1 will have a protein strongly enriched in the bouton border proximity. The bouton on the right side has a redistribution ≤1 and will have a protein equally distributed in the bouton border proximity, as well as within 700 nm for the border. This quantification was also used to determine the redistribution of Lifeact upon YARS1 expression (Fig. 4a). (**b)** Neuronal overexpression of the actin-bundling protein PLS3 rearranged presynaptic F-actin from bouton border (arrows) inwards, as quantified in (**c)**. Error bars – SEM; ** p=0.0078, two-sided unpaired t-test. The control NMJ is reused from Fig. 4a. (**d)** Representative NMJ and bouton from larvae co-expressing GFP-tagged Act5C as an independent marker with YARS1^WT^, YARS1^E196K^ and PLS3 demonstrate decrease in the number of presynaptic actin assemblies compared to controls (CD8::RFP). Scale bars – 10 µm NMJ image, 2 µm bouton image. (**e)** Quantification of the number of Act5C::GFP positive actin assemblies in boutons. N is individual actin assemblies (between 42 and 78, extracted form 12 NMJs from 6 larvae per genotype). Error bars – SEM. *** p=0.0004, p=0.0003, and p<0.0001 for YARS^WT^, YARS^E196K^ and PLS3 respectively, after a two-sided unpaired t-test. Source data are provided as a Source data file.


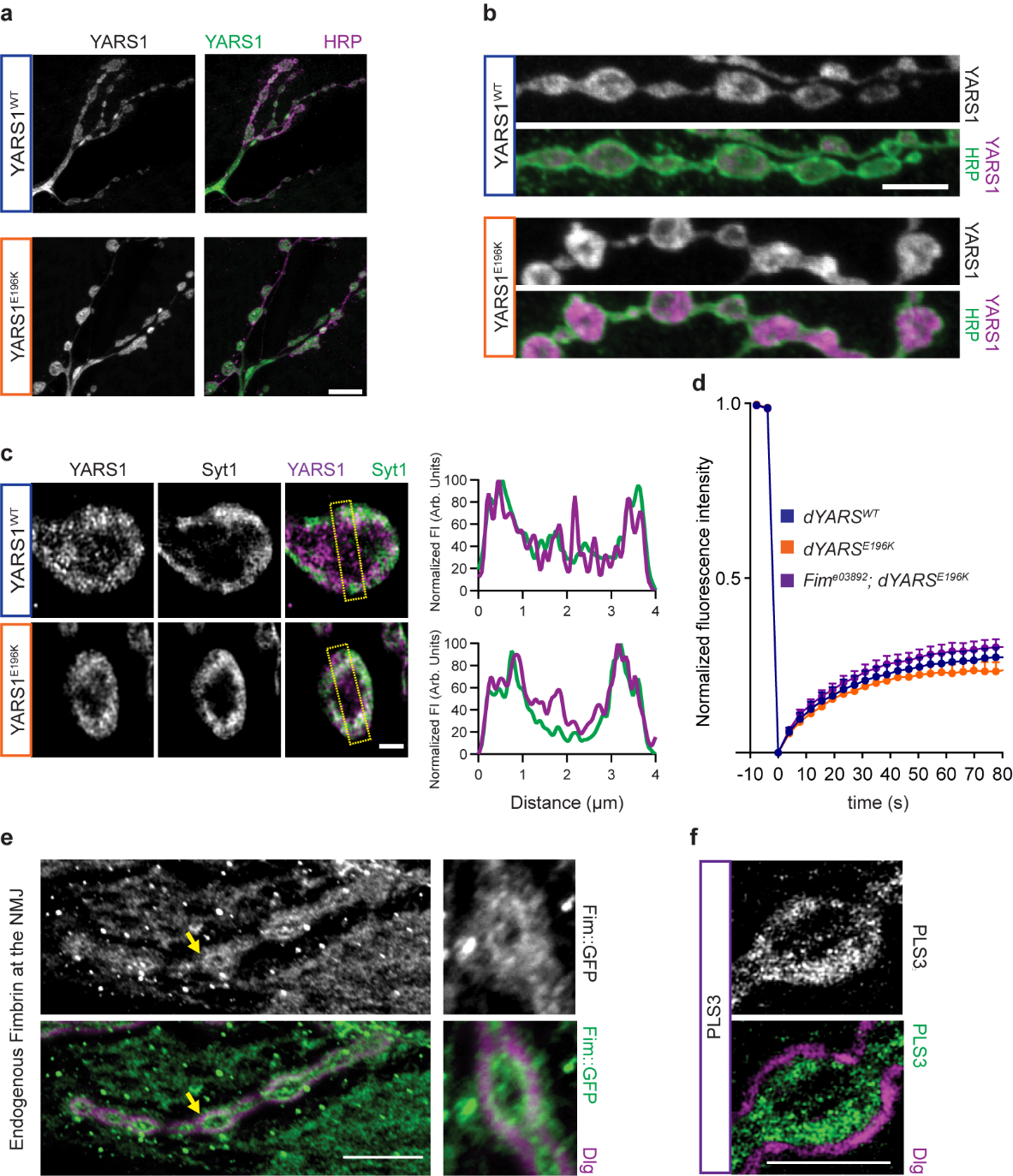


**Supplementary Figure 9. Assessment of the presynaptic localization of YARS1 and Fimbrin/Plastin and their effects on synaptic function at the *Drosophila* larval NMJs.**

**(a)** Localization of immunolabelled YARS1 (green) at the NMJ in larvae expressing WT- and CMT YARS1^E196K^ pan-neuronally (*nSyb-Gal4*), with neuronal membrane marker HRP in magenta. Scale bar – 10 µm. (**b)** Closer look at presynaptic boutons of the two genotypes with the same fluorescent signal labelling. Scale bar – 10 µm. **(c)** SIM images of boutons at the larval NMJs depicting YARS1 (magenta) and synaptic vesicles (Syt::eGFP – green), with plotted FI intensities on right. Scale bar – 2 µm. (**d)** Fluorescence intensity recovery curves of the Syt::eGFP signal after photobleaching (see Fig. 5c) in the three marked genotypes; n = 19 (*dYARS^WT^*), 13 (*dYARS^E196K^*), and 29 (*Fim^e03892^; dYARS^E196K^*) individual boutons from larvae from at least three independent experiments. The symbols represent the mean value for each time point; corresponding FRAP sequence images and quantification of the mobile fraction are shown in Fig. 5c, d, accordingly. Error bars – SEM. (**e)** GFP protein trap line of Fimbrin (Fim::GFP) depicts endogenous Fimbrin at the NMJ in boutons (yellow arrow/inset), in the subsynaptic reticulum area around the bouton overlapping with the postsynaptic Dlg signal, and in muscles of third instar larvae. (**f)** SIM image of immunolabelled PLS3 (green) and Dlg (magenta) in a bouton of *nSyb-Gal4>UAS-PLS3* larva. The experiments were repeated at least three independent times with similar outcomes. Source data are provided as a Source data file.


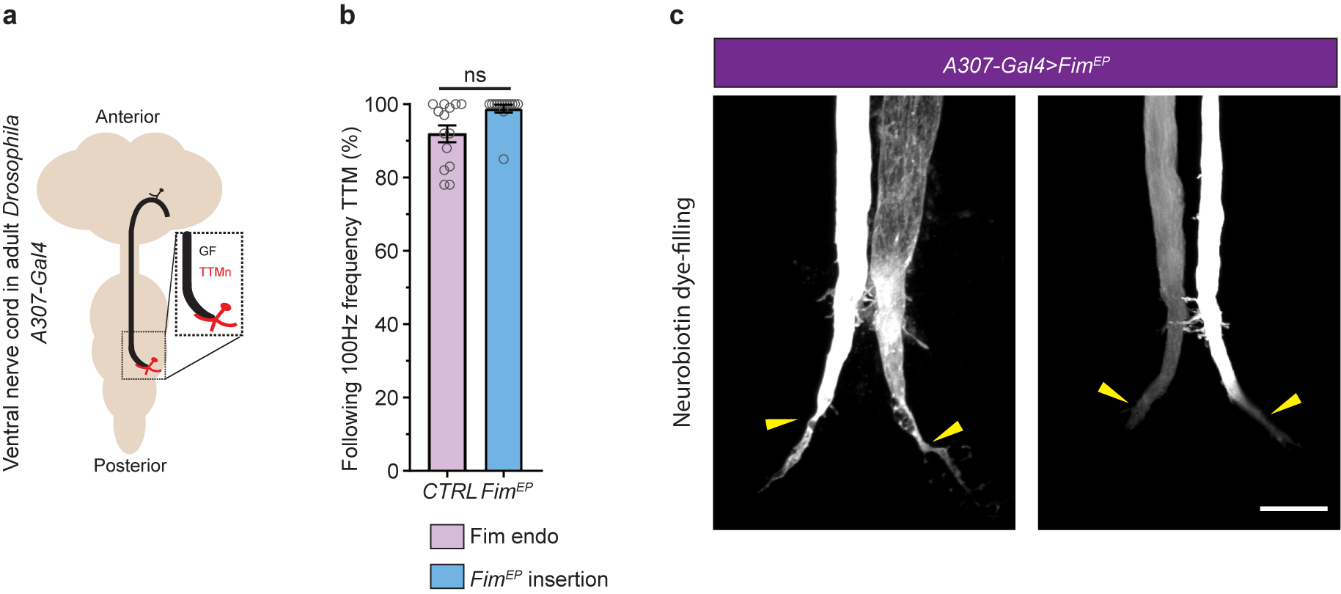


**Supplementary Figure 10. Assessment of the function and morphology of the giant fibers in a *Fim^EP^* adult flies.**

**(a)** A scheme of the GF-TTMn synapse. (**b)** High frequency stimulations of the GF-TTMn synapse at 100 Hz in control flies (*A307-Gal4/+*) and flies carrying the *Fim^EP^* insertion in absence of *A307-Gal4*; *n* = 14 (both genotypes) individual giant fiber recordings from eight days-old female flies (**c)** GF terminal morphology visualized by neurobiotin dye injection in *A307-Gal4>Fim^EP^* expressing flies with abnormal electrophysiology. Thinning of the axon terminals, as well as shorter terminals and constrictions were observed in these flies (yellow arrows). n≥10 recordings from at least 6 flies per genotype. Error bars – SEM. Ns, non-significant after two-sided unpaired t-test. Scale bar – 50 µm. Source data are provided as a Source data file.


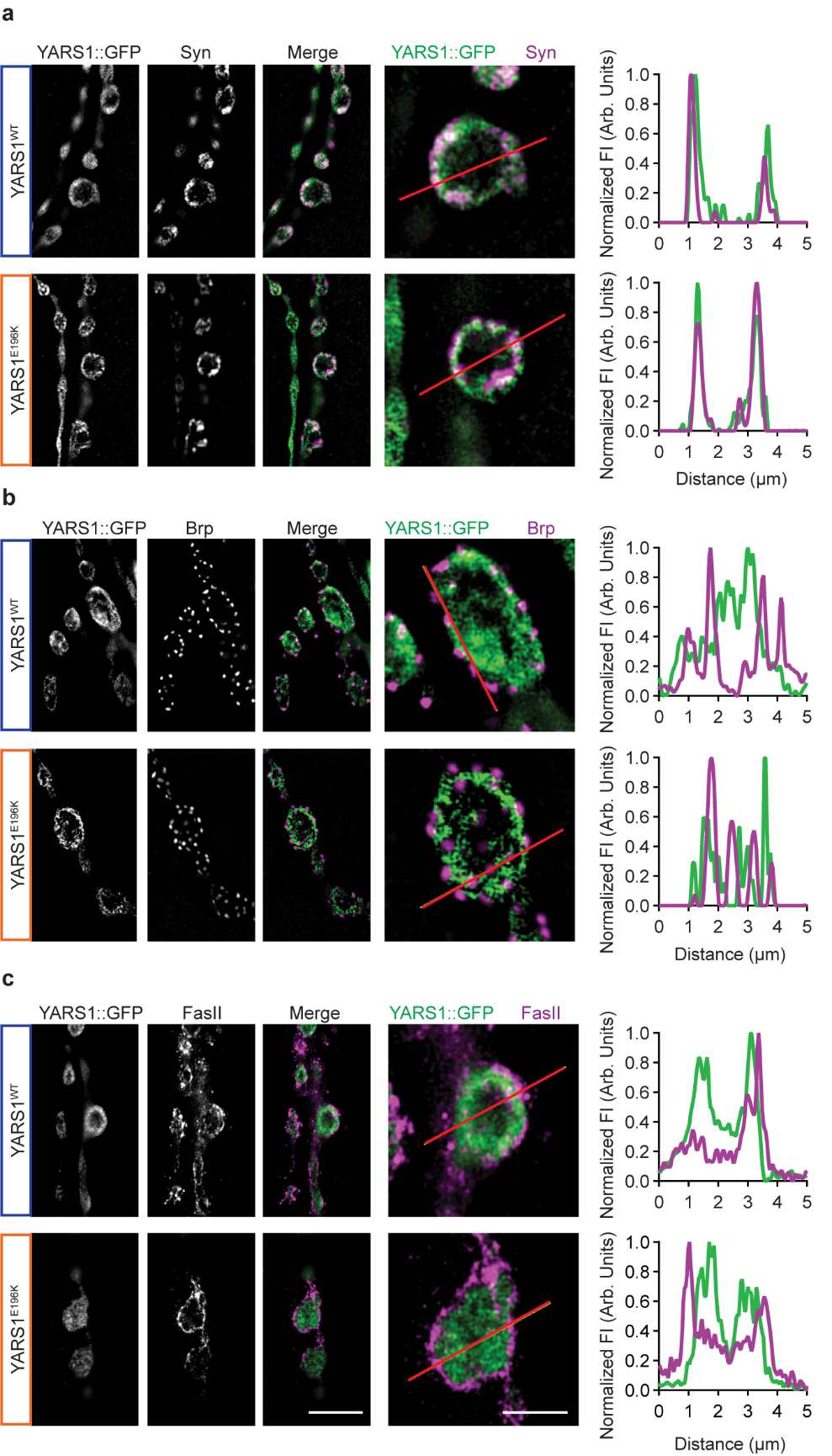


**Supplementary Figure 11. Co-localization of YARS1 with synaptic markers at the NMJs.**

**(a)** Structured Illumination Microscopy (SIM) images of immunolabeled YARS1::GFP demonstrate co-localization with the SV-F-actin binding protein Synapsin in the boutons, upon *nSyb-Gal4*-driven expression of wild type and mutant *YARS1*. (**b)** No major overlap was detected between YARS1 and the active zone protein Brp. Of note, the line is drawn at the bouton periphery in order to capture as many active zones as possible. (**c)** No major overlap with the periactive zone protein FasII. All images represent single slices. Scale bars – 5 µm in the image representing the larger portion of the NMJ, and 2 µm on the zoomed bouton on which plot line was drawn. The experiments were repeated at least three independent times with similar outcomes.

**References**

1 Murali, T. *et al.* DroID 2011: a comprehensive, integrated resource for protein, transcription factor, RNA and gene interactions for Drosophila. *Nucleic Acids Res* **39**, D736-743, doi:10.1093/nar/gkq1092 (2011).

2 Sievers, F. *et al.* Fast, scalable generation of high-quality protein multiple sequence alignments using Clustal Omega. *Mol Syst Biol* **7**, 539, doi:10.1038/msb.2011.75 (2011).

3 Shinomiya, H. Plastin family of actin-bundling proteins: its functions in leukocytes, neurons, intestines, and cancer. *Int J Cell Biol* **2012**, 213492-213492, doi:10.1155/2012/213492 (2012).

4 Oprea, G. E. *et al.* Plastin 3 is a protective modifier of autosomal recessive spinal muscular atrophy. *Science* **320**, 524-527, doi:10.1126/science.1155085 (2008).

5 Hosseinibarkooie, S., Schneider, S. & Wirth, B. Advances in understanding the role of disease-associated proteins in spinal muscular atrophy. *Expert Rev Proteomics* **14**, 581-592, doi:10.1080/14789450.2017.1345631 (2017).
