# Supplementary material for: Tyrosyl-tRNA synthetase has a noncanonical function in actin bundling
Source: Nat Commun. 2023 Mar 8;14:999. doi: 10.1038/s41467-023-35908-3 (PMC9995517; doi:10.1038/s41467-023-35908-3)
Supplement: Supplementary file 3 — Description of Additional Supplementary Files [file 41467_2023_35908_MOESM3_ESM.pdf]

Description of Additional Supplementary Files

File Name: Supplementary Data 1
Description: X-chromosome-associated *EP* lines used for a retinal degeneration screen in YARS1^{E196K} Drosophila model.

File Name: Supplementary Data 2
Description: Table of mass-spectrometry data in FLAG-YARS1WT expressing HEK293 cells.

File Name: Supplementary Movie 1
Description: TIRF movies of unanchored actin filaments in the presence of YARS1 proteins.

File Name: Supplementary Movie 2
Description: YARS1^{WT} and F-actin at protrusions in HeLa cells.
