# Supplementary material for: Tyrosyl-tRNA synthetase has a noncanonical function in actin bundling
Source: Nat Commun. 2023 Mar 8;14:999. doi: 10.1038/s41467-023-35908-3 (PMC9995517; doi:10.1038/s41467-023-35908-3)
Supplement: Supplementary file 8 — Reporting Summary [file 41467_2023_35908_MOESM8_ESM.pdf]

nature portfolio

Corresponding author(s): Albena Jordanova, PhD

Last updated by author(s): 2022/12/21

Reporting Summary

Nature Portfolio wishes to improve the reproducibility of the work that we publish. This form provides structure for consistency and transparency in reporting. For further information on Nature Portfolio policies, see our Editorial Policies and the Editorial Policy Checklist.

Statistics

For all statistical analyses, confirm that the following items are present in the figure legend, table legend, main text, or Methods section.

n/a	Confirmed	
☐	☒	The exact sample size (*n*) for each experimental group/condition, given as a discrete number and unit of measurement
☐	☒	A statement on whether measurements were taken from distinct samples or whether the same sample was measured repeatedly
☐	☒	The statistical test(s) used AND whether they are one- or two-sided<br>*Only common tests should be described solely by name; describe more complex techniques in the Methods section.*
☒	☐	A description of all covariates tested
☐	☒	A description of any assumptions or corrections, such as tests of normality and adjustment for multiple comparisons
☐	☒	A full description of the statistical parameters including central tendency (e.g. means) or other basic estimates (e.g. regression coefficient) AND variation (e.g. standard deviation) or associated estimates of uncertainty (e.g. confidence intervals)
☐	☒	For null hypothesis testing, the test statistic (e.g. *F*, *t*, *r*) with confidence intervals, effect sizes, degrees of freedom and *P* value noted<br>*Give P values as exact values whenever suitable.*
☒	☐	For Bayesian analysis, information on the choice of priors and Markov chain Monte Carlo settings
☒	☐	For hierarchical and complex designs, identification of the appropriate level for tests and full reporting of outcomes
☒	☐	Estimates of effect sizes (e.g. Cohen's *d*, Pearson's *r*), indicating how they were calculated

Our web collection on statistics for biologists contains articles on many of the points above.

Software and code

Policy information about availability of computer code

Data collection

Scanning electron micrographs of adult fly eyes were captured on SEM505 microscope (Philips).
Western blot membranes were imaged on ImageQuantTM LAS 4000 (GE Healthcare Life Sciences) and AI600 chemiluminescent imager (GE Healthcare). Coomassie solution stained gels were imaged on a ChemiDoc Imaging system (Bio-Rad).
TIRF movies were imaged on Nikon-Ti200 inverted microscope equipped with a 150 mW Argon Laser (Mellot Griot, Carlsbad, CA), a TIRF-objective with NA of 1.49 (Nikon Instruments Inc., New York, NY) and an EMCCD camera (Andor Ixon, Belfast, Northern Ireland), using the optimal focus via the perfect focus system.
Fibroblasts were imaged using laser scanning confocal microscope Carl Zeiss LSM700 equipped with a 40x (1.3 NA) Plan-Neofluar objective.
Cell migration assay in SH-SY5Y cells was measured using the Incucyte® Cell Migration Kit (Sartorius). Phase contrast imaging of SH-SY5Y cells was done on a Zeiss Axiovert 200M microscope equipped with a Zeiss AxioCam MR3 camera and a LCI Plan-Neofluar 63x/1.30 Imm Korr Ph 3 objective. Phase-contrast imaging to assess projections was done on a Zeiss Axiovert 200M microscope equipped with a Zeiss AxioCam MR3 camera and 20x phase contrast objective.
Carl Zeiss LSM700 microscope equipped with a 63x Plan-Apochromat (1.4 NA) objective was used to image Lifeact signal at the Drosophila NMJs.
Intracellular recordings from the tergotrochanteral muscle (TTM) of adult flies were performed after activation with 0.03 ms pulses of 30-60 V using two tungsten electrodes inserted into the brain (Grass S44 stimulator, Grass Instruments). The recordings were amplified (Getting 5A amplifier, Getting Instruments) and the signals were stored and analyzed using pCLAMP software (Molecular Devices).
Neurobiotin-filled giant fiber axon terminal image acquisition was performed on Nikon C1si Fast Spectral Confocal system using a 60× oil immersion objective lens was used to image giant fibers.
FRAP was performed on an inverted Zeiss LSM700 confocal laser scanning microscope using a 63x Plan-Apochromat objective (1.4 NA) and controlled by Zen2009 software.

Structured illumination microscopy of larval NMJs was performed on a Zeiss ELYRA S.1 microscope equipped with a 63x Plan-Apochromat objective (1.4 NA).
Act5C::GFP signal at the larval NMJs was acquired using a Nikon Ni-E upright microscope equipped with a Yokogawa CSU-W1 spinning-disk head, an Andor iXon 897U EMCCD camera and Nikon Elements AR software. A 60x (NA 1.4) oil immersion objective.
Trypsin digested peptides from the FLAG-YARS1 immunoprecipitation were analyzed using a Q Exactive Orbitrap mass spectrometer (Thermo Fisher).
Advancing versions of ImageJ from 1.45a (6 February 2011) including the latest 1.53t (24 August 2022) were used in the study (ImageJ (nih.gov)). The EP-targeted genes' annotations, expression data and functional annotations were retrieved from advancing versions of FlyBase, including the latest FB2022_06 (FlyBase Homepage). We used the PANTHER Classification System, version 16.0 to retrieve gene ontology (GO) information (pantherdb.org). The quantitative proteomics software package MaxQuant version 1.5.3.8 was used to obtain label-free quantification (LFQ) from the mass-spec data (MaxQuant). ClustalOmega Version 1.2.2 was used for protein sequence alignment (Clustal Omega < Multiple Sequence Alignment < EMBL-EBI). DroID data base version DroID_v2018_08 (DroID: The Comprehensive Drosophila Interactions Database (droidb.org)).

Data analysis

A separate "Data availability" section is provided in the manuscript.
All data generated and/or analysed during this study are included in this article (and its supplementary files). The source data underlying Figures 2, 3, 4, 5 and 6, and Supplementary Figures 1, 2, 4, 5, 7, 8, 9, and 10 are provided as a Source Data file. The mass spectrometry data are deposited at the PRIDE database (PXD037630). All other relevant data (e.g. ImageJ macro scripts) are available from the corresponding author on reasonable request. The human biomaterials are available subject to MTA. Databases and softwares relevant to the study include (ImageJ (nih.gov)); FlyBase, (FlyBase Homepage); PANTHER Classification System (pantherdb.org); the quantitative proteomics software package MaxQuant (MaxQuant); ClustalOmega (Clustal Omega < Multiple Sequence Alignment < EMBL-EBI); DroID data base DroID: The Comprehensive Drosophila Interactions Database (droidb.org)).

For manuscripts utilizing custom algorithms or software that are central to the research but not yet described in published literature, software must be made available to editors and reviewers. We strongly encourage code deposition in a community repository (e.g. GitHub). See the Nature Portfolio guidelines for submitting code & software for further information.

Data

Policy information about availability of data

All manuscripts must include a data availability statement. This statement should provide the following information, where applicable:

- Accession codes, unique identifiers, or web links for publicly available datasets
- A description of any restrictions on data availability
- For clinical datasets or third party data, please ensure that the statement adheres to our policy

A separate "Data availability" section is provided in the manuscript.
All data generated and/or analysed during this study are included in this article (and its supplementary files). The source data underlying Figures 2, 3, 4, 5 and 6, and Supplementary Figures 1, 2, 4, 5, 7, 8, 9, and 10 are provided as a Source Data file. The mass spectrometry data are deposited at the PRIDE database (PXD037630). All other relevant data are available from the corresponding author on reasonable request. The human biomaterials are available subject to MTA. Databases and softwares relevant to the study include (ImageJ (nih.gov)); FlyBase, (FlyBase Homepage); PANTHER Classification System (pantherdb.org); the quantitative proteomics software package MaxQuant (MaxQuant); ClustalOmega (Clustal Omega < Multiple Sequence Alignment < EMBL-EBI); DroID data base DroID: The Comprehensive Drosophila Interactions Database (droidb.org)).
A separate "Code availability" section is provided in the manuscript as well, stating that "Relevant code and classifiers (e.g. ImageJ macro scipts) are available from the corresponding author on reasonable request".

Human research participants

Policy information about studies involving human research participants and Sex and Gender in Research.

Reporting on sex and gender

The findings apply for both sexes. No individuals level information was reported.

Population characteristics

Dermal fibroblasts from one YARS1-E196K/WT patient (male, 44 y) and one control individual (male, 42 y) were sampled using standard procedures and after obtaining their written informed consent. The study complies with the ethical guidelines of the Medical University-Sofia, Bulgaria and University of Antwerp, Belgium and was approved by the respective local institutional review boards.

Recruitment

The CMT patient was recruited upon clinical examination by Dr. Ivaylo Tournev who is an expert neurologist at the Medical University-Sofia, Bulgaria. Dr. Tournev is included as a co-author in this manuscript. The subtype of CMT caused by the dominant YARS1-E196K mutation is rare and limited to a single large pedigree from Bulgaria. This might present a bias however we do not have evidence to either prove of disprove this at the moment.

Ethics oversight

We have complied with all relevant ethical regulations for animal and human testing and research. The study was approved by the Ethical committee of the University of Antwerp and University Hospital Antwerp (14/15/188). Informed consent was obtained from all individuals included in the study.

Note that full information on the approval of the study protocol must also be provided in the manuscript.

Field-specific reporting

Please select the one below that is the best fit for your research. If you are not sure, read the appropriate sections before making your selection.

- [x] Life sciences
- [ ] Behavioural & social sciences
- [ ] Ecological, evolutionary & environmental sciences

For a reference copy of the document with all sections, see nature.com/documents/nr-reporting-summary-flat.pdf

Life sciences study design

All studies must disclose on these points even when the disclosure is negative.

Sample size	No sample size determination was performed. Sample sizes were chosen based on our own previous experience and based on sample sizes reported in the literature. The sample sizes regarding the Drosophila experiments are higher than 10 measurements when possible and come from at least five different animals. The sample size was up scaled, when possible, for example in the experiments performed in cell lines. Sample sizes are stated in the legends of all figures.
Data exclusions	From the FRAP experiment we excluded boutons with poor curve fitting parameter ($R^2 < 0.95$). From cell migration experiment in SH-SY5Y cells low-quality wounds (irregular, occurrence of cell debris) were removed (<10% of wells).
Replication	In the retinal degeneration screen at least 20 female flies from the control and test genotypes were compared with each other. Genetic crosses for positive hits were repeated at least three independent times. Other Drosophila-related experiments also were performed on larvae or adults coming from at least three independent genetic crosses. The in vitro pelleting assays were performed at least three independent times, except for HARS1 (two times). TIRF experiments were performed two independent times at two different concentrations. Each figure contains information in the legend on the independent experiments performed. All attempts at replication were successful. As we describe in the Methods section, the TIRF experiments were repeated several times and in each case F-actin bundle and cable formation was induced by YARS1; however, there was frequent "stickiness" observed in the presence of YARS1 that prevented robust scoring of real-time events with TIRF.
Randomization	In the experiments involving Drosophila, experimental groups were formed by choosing animals with the correct genotype (usually controlled by the absence or presence of genetic markers), at the appropriate developmental stage (adult or third instar wandering larvae), and gender (females). In the cellular experiments and TIRF, imaging was randomized to different parts of the slide.
Blinding	Many of our studies involved unbiased way of quantification of the phenomena thus we did use blinding.

Reporting for specific materials, systems and methods

We require information from authors about some types of materials, experimental systems and methods used in many studies. Here, indicate whether each material, system or method listed is relevant to your study. If you are not sure if a list item applies to your research, read the appropriate section before selecting a response.

Materials & experimental systems

n/a	Involved in the study	
[ ]	[x]	Antibodies
[ ]	[x]	Eukaryotic cell lines
[x]	[ ]	Palaeontology and archaeology
[ ]	[x]	Animals and other organisms
[x]	[ ]	Clinical data
[x]	[ ]	Dual use research of concern

Methods

n/a	Involved in the study	
[x]	[ ]	ChIP-seq
[x]	[ ]	Flow cytometry
[x]	[ ]	MRI-based neuroimaging

Antibodies

Antibodies used

Alexa Fluor® 488 AffiniPure Goat Polyclonal Anti-Horseradish Peroxidase (Jackson Immunoresearch), catalog number 123-545-021
Rhodamine Red™-X (RRX) AffiniPure Goat Polyclonal Anti-Horseradish Peroxidase (Jackson Immunoresearch), catalog number 123-295-021
Mouse monoclonal DLG antibody: Developmental Studies Hybridoma Bank (DSHB) , catalog number: 4F3 anti-discs large.
Mouse monoclonal Brp: DHSB, nc82.
Mouse monoclonal FasII: DHSB, 1D4.
Mouse monoclonal Synapsin: xDHSB, SynORF1, 3C11.
Mouse monoclonal TyrRS antibody: Abnova, catalog number: H00008565-M02.
Mouse monoclonal α-Tubulin antibody: Abcam, catalog number: ab7291.
Rabbit monoclonal Flag antibody: Sigma-Aldrich, catalog number: F2555.
Rabbit polyclonal to T-Plastin/PLS3: Abcam ab137585.

	Rabbit polyclonal to L-plastin/PLS2: Abcam ab83496; product not available at the moment.<br>Rabbit polyclonal GFP: Invitrogen Catalog number A-11122.<br>Rabbit polyclonal RFP: Abcam (ab62341).<br>α-GFP nanobodies (Nanotag Biotechnologies)<br>Alexa Fluor®-488 and Alexa Fluor®-546 secondary antibodies (Invitrogen)<br>AlexaFluorTM 594 Phalloidin (Thermo Fisher Scientific)<br>Streptavidin-Cy2 conjugate (Jackson ImmunoResearch)<br>10% w/v neurobiotin (Vector Labs)<br>Tetramethyl rhodamine-labeled dextran (Invitrogen)
Validation	Alexa Fluor® 488 AffiniPure Goat Polyclonal Anti-Horseradish Peroxidase (Jackson Immunoresearch), catalog number 123-545-021 Alexa Fluor® 488-Goat Anti-Peroxidase Antibody | JIR (jacksonimmuno.com)<br>Rhodamine Red™-X (RRX) AffiniPure Goat Polyclonal Anti-Horseradish Peroxidase (Jackson Immunoresearch), catalog number 123-295-021 RR-X-AffiniPure Goat Anti-Peroxidase Antibody | JIR (jacksonimmuno.com)<br>Mouse monoclonal DLG antibody: Developmental Studies Hybridoma Bank, catalog number: 4F3 anti-discs large. Referenced in: PMID: 15556867; PMID: 24772085<br>Mouse monoclonal Brp (nc82, DHSB). Referenced in: PMID: 36103878; PMID: 36367867<br>Mouse monoclonal FasII (1D4, DHSB). Referenced in: PMID: 33729157, PMID: 32628110<br>Mouse monoclonal Synapsin (SynORF1, 3C11, DHSB). Referenced: PMID: 32882234; PMID: 29728423<br>Mouse monoclonal TyrRS antibody: Abnova, catalog number: H00008565-M02. Referenced: PMID: 26138142; PMID: 19561293<br>Mouse monoclonal α-Tubulin antibody: abcam, catalog number: ab7291. Referenced: PMID: 34190355; PMID: 33813791<br>Rabbit monoclonal Flag antibody: Sigma-Aldrich, catalog number: F2555; Referenced in: PMID: 29179207; PMID: 25847407<br>Rabbit polyclonal to T-Plastin/PLS3: Abcam ab137585. Referenced in: PMID: 27358069<br>Rabbit polyclonal to L-plastin/PLS2: Abcam ab83496; product not available at the moment. Reference in: PMID: 30479022<br>Rabbit polyclonal GFP: Invitrogen Catalog number A-11122; PMID: 30854433; PMID: 32366843; PMID: 30111579<br>Rabbit polyclonal RFP: Abcam (ab62341). PMID: 33075563; PMID: 33237838; PMID: 33531476

Eukaryotic cell lines

Policy information about cell lines and Sex and Gender in Research

Cell line source(s)	HEK293T cell line (ACC 635) and HELA cell line (ACC 57) were obtained from the German Collection of Microorganisms and Cell Cultures GmbH (DSMZ) at the Leibniz Institute.<br>The HEK293T are highly transfectable derivative of the human primary embryonal kidney cell line 293 (ACC 305). Their morphology matches the DSMZ description as oval to fibroblastoid cells growing adherently as monolayer.<br>The HELA cell line originates from the epitheloid cervix carcinoma of a 31-year-old woman in 1951, later re-diagnosed with adenocarcinoma. The cells' morphology matches the DSMZ description as epithelial-like cells growing in monolayers.<br>Human dermal fibroblasts were derived from skin biopsies from control individuals and a CMT patient carrying the YARS1-E196K mutation.<br>SH-SY5Y (CRL 2266) cell line was purchased from ATCC and it is a thrice cloned subline of the neuroblastoma cell line SK-N-SH (ATCC HTB-11), which was established in 1970 from a metastatic bone tumor from a 4-year-old cancer patient.
Authentication	Authentication of the patient's fibroblasts was performed by haplotype and Sanger sequencing analyzes using the information published in PMID: 16429158 and PMID: 14606043.<br>The HEK293T authenticity was confirmed at DSMZ via STR analysis according to the global standard ANSI/ATCC ASN-0002.1-2021 (2021). Cytogenetics confirmed human flat-moded near-triploid karyotype.<br>The HELA cell line authenticity was confirmed at DSMZ by STR analysis according to the global standard ANSI/ATCC ASN-0002.1-2021 (2021). Cytogenetics show human hypertriploid/hypotetraploid karyotype with 15% polyploidy.<br>SH-SY5Y (CRL 2266) cell line authenticity assessed at ATCC via STR profiling (Amelogenin: X; CSF1PO: 11; D13S317: 11; D16S539: 8,13; D5S818: 12; D7S820: 7,10; THO1: 7,10; TPOX: 8,11; vWA: 14,18).
Mycoplasma contamination	All cell lines tested negative for mycoplasma contamination.
Commonly misidentified lines<br>(See ICLAC register)	None of the lines used are commonly misidentified.

Animals and other research organisms

Policy information about studies involving animals; ARRIVE guidelines recommended for reporting animal research, and Sex and Gender in Research

Laboratory animals	Drosophila melanogaster animals were used throughout the study. Female flies were tested in all experiments, unless otherwise specified. Virgin female flies (1-2 weeks old) were crossed to male flies with unspecified age. In the eye screen, ommatidial disorganization was assessed in female flies aged 1-2 weeks. For the giant fiber recordings and dye-fills, 8 days-old aged-matched female flies were used (also specified in the main text). All NMJ experiments were performed in third instar wandering larvae (also specified throughout the main text). All transgenic flies used are in the w1118 genetic background.
Wild animals	No wild animals were used in this study.

Reporting on sex	Female adult flies were predominantly used throughout the study, unless otherwise stated. Gender is easily assigned in most stages of the Drosophila life cycle based on distinct anatomy features.
Field-collected samples	This work did not involve field-collected samples.
Ethics oversight	Research on Drosophila does not require ethical clearance according to the regulations of the University of Antwerp, Belgium.

Note that full information on the approval of the study protocol must also be provided in the manuscript.
